# A review of health utilities using the EQ-5D in studies of cardiovascular disease

**DOI:** 10.1186/1477-7525-8-13

**Published:** 2010-01-28

**Authors:** Matthew TD Dyer, Kimberley A Goldsmith, Linda S Sharples, Martin J Buxton

**Affiliations:** 1 Health Economics Research Group, Brunel University, Uxbridge, UK; 2 Papworth Hospital NHS Trust, Cambridge UK; 3 MRC Biostatistics Unit, Institute of Public Health, Cambridge, UK; 4 National Collaborating Centre for Mental Health, The Royal College of Psychiatrists, London, UK

## Abstract

**Background:**

The EQ-5D has been extensively used to assess patient utility in trials of new treatments within the cardiovascular field. The aims of this study were to review evidence of the validity and reliability of the EQ-5D, and to summarise utility scores based on the use of the EQ-5D in clinical trials and in studies of patients with cardiovascular disease.

**Methods:**

A structured literature search was conducted using keywords related to cardiovascular disease and EQ-5D. Original research studies of patients with cardiovascular disease that reported EQ-5D results and its measurement properties were included.

**Results:**

Of 147 identified papers, 66 met the selection criteria, with 10 studies reporting evidence on validity or reliability and 60 reporting EQ-5D responses (VAS or self-classification). Mean EQ-5D index-based scores ranged from 0.24 (SD 0.39) to 0.90 (SD 0.16), while VAS scores ranged from 37 (SD 21) to 89 (no SD reported). Stratification of EQ-5D index scores by disease severity revealed that scores decreased from a mean of 0.78 (SD 0.18) to 0.51 (SD 0.21) for mild to severe disease in heart failure patients and from 0.80 (SD 0.05) to 0.45 (SD 0.22) for mild to severe disease in angina patients.

**Conclusions:**

The published evidence generally supports the validity and reliability of the EQ-5D as an outcome measure within the cardiovascular area. This review provides utility estimates across a range of cardiovascular subgroups and treatments that may be useful for future modelling of utilities and QALYs in economic evaluations within the cardiovascular area.

## Background

Cardiovascular disease (CVD) imposes a great burden on societies around the world, with an estimated 16.7 million - or 29.2% of total global deaths - resulting from various forms of CVD[1]. A recent study estimated the total costs of CVD in the European Union, in terms of health care expenditure and lost productivity, to be €169bn a year [2]. Major CVDs include coronary heart disease (CHD), cerebrovascular disease, hypertension and heart failure. In addition, CVD has a significant impact on health-related quality of life (HRQoL) in patients who survive coronary events such as heart attacks (myocardial infarction) or stroke. It has been suggested that HRQoL measures (i.e. measures that refer to a patient's emotional, social and physical wellbeing) are particularly useful with respect to investigating treatment of CVD in three instances: 1) when results of clinical trials show little evidence of a major improvement in survival so that choice of therapy will be determined on the basis of quality of life measurement; 2) when a treatment is effective in reducing mortality, but has toxic or unacceptable side effects so that quality of life measurement may help physicians and their patients weight the benefits and risks of such a treatment; 3) when patients are asymptomatic or have mild symptoms, the morbidity and mortality rates are low, and the therapy is long term[3].

Increasingly over time, clinical trials within the cardiovascular field have included HRQoL measures. Such measures, alongside clinical measures of functionality, can help evaluate the physical, mental and emotional implications of CVD as well as the effects of surgical and medical treatments. Commonly used functional classification systems within the cardiovascular field are the New York Heart Association (NYHA) functional classification system for heart failure patients and the Canadian Cardiovascular Society (CCS) grading scale for angina pectoris[4,5]. HRQoL measurement in CVD can be assessed using disease-specific instruments such as the Seattle Angina Questionnaire (SAQ); MacNew Heart Disease Health-related Quality of Life Questionnaire; and the Minnesota Living with Heart Failure score (MLHF) [6-8]. These questionnaires are particularly sensitive to changes in aspects of HRQoL directly related to CVD. Alternatively, commonly used generic measures of HRQoL including the SF-6D, Health Utilities Index (HUI) and the EQ-5D have also been used in CVD studies [9-11]. The main advantages of such generic multi-attribute health state classifiers are that they allow the calculation of Quality adjusted life years (QALYs) within cost-utility analyses as well as allowing comparison of HRQoL across different conditions and against age-sex matched population norms.

Among the available generic measures, the EQ-5D has gained widespread use due to its simplicity to administer, score and interpret. It also imposes minimal burden on the respondent as it is a brief, simple measure for patients to understand and to complete. The index-based score is generated by applying societal preference weights to the health state classification completed by the patient that consists of five dimensions (mobility, self-care, usual activities, pain/discomfort, and anxiety/depression), each with three levels of response or severity (no problems, some problems, or extreme problems). The ability to convert self classification responses into a single index score makes the EQ-5D practical for clinical and economic evaluation[11]. The index-based score is typically interpreted along a scale where 1 represents best possible health and 0 represents dead, with some health states valued as being worse than dead (<0). In addition to the index-based scoring system, the visual analogue scale (VAS) component of the EQ-5D enables the patient to place their current health state on a range from 0 (worst imaginable health state) to 100 (best imaginable health state). Algorithms have been developed based on societal preferences for health states, with the most popular being based on the UK-based population[12], although many other country-specific algorithms are also available [13-18].

The principle aims of this paper were: to synthesise the evidence on the validity and reliability of the EQ-5D in studies within the cardiovascular field; to summarise the EQ-5D based scores reported in studies within the CVD field; and to attempt to stratify mean utility scores according to level of disease severity.

## Methods

### Data Collection and Assessment

A computerised search of the current published literature was performed using MEDLINE and EMBASE for the period January 1988 to October 2008. The search strategy combined exploded or medical subject headings relating to the CVD field and the EQ-5D as follows: ('cardiovascular'/exp OR 'cardiovascular') OR ('cardiac'/exp OR 'cardiac') OR ('cardiology'/exp OR 'cardiology') AND 'euroqol' OR 'EQ 5D' OR 'EQ5D'. The EuroQol website http://www.euroqol.org was also used to identify unique references, including working papers and conference proceedings that may not have been captured in the initial literature search. Only full-text published papers were included for analysis.

**Figure 1:**
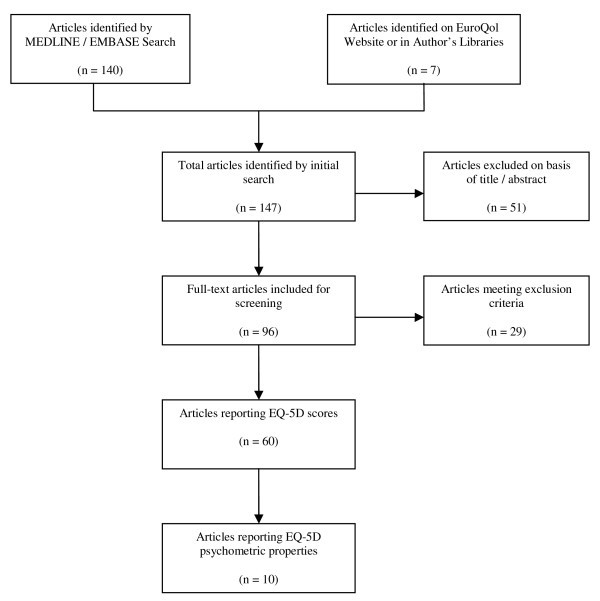
**Summary of literature search**.

The inclusion criteria required that the paper was original research, and that it reported EQ-5D scores specific to cardiovascular disease or reported psychometric properties of the EQ-5D in a population with cardiovascular disease. Studies that only reported EQ-5D index or VAS scores graphically in terms of change over time were excluded from the analysis. When multiple studies used the same dataset, EQ-5D scores were only reported from one article to avoid double counting. No language restrictions were imposed. Study abstracts that potentially met the inclusion criteria were identified, and full-text articles were retrieved for further review. A standard data abstraction form was developed to facilitate the structured review, which included study design, patient characteristics, intervention information, published source of index-based preference weights and EQ-5D scores as well as details of any other clinical measures; disease-specific quality of life and generic HRQoL instruments. A summary of the results of the literature search is provided in figure 1.

### Data Analysis

**Figure 2:**
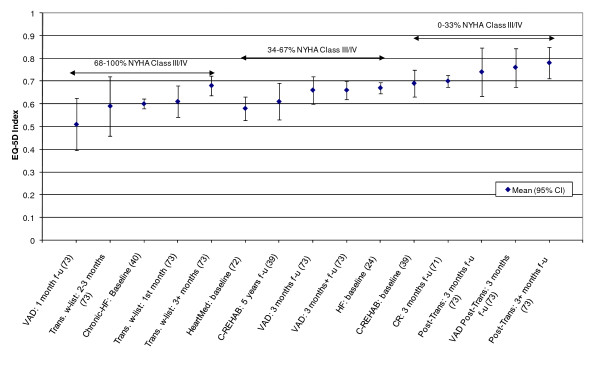
**EQ-5D Index Mean scores for Heart Failure patients - Stratified by baseline disease severity (NYHA class)**.

**Figure 3:**
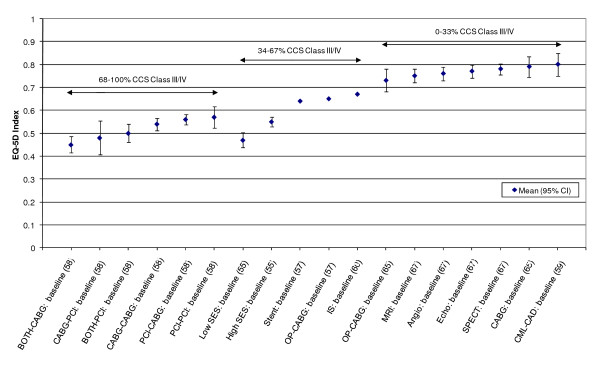
**EQ-5D Index Mean scores for IHD patients - Stratified by baseline disease severity (CCS class)**.

Initially, studies that reported EQ-5D index-based scores and/or VAS scores were sorted into cardiovascular subgroups (e.g. Angina/Myocardial Infarction/CHD etc) that were informed by the latest WHO International Classification of Diseases (ICD-10: I00-I99 - diseases of the circulatory system) (Table 1 in Additional file 1) [19]. Confidence intervals were calculated from the sample size and standard deviation (SD) or the standard error when not reported directly in the paper. Scores that were not reported using the appropriate range of scale were transformed, i.e. index-based scores anchored by 0 (dead) and 1 (full health), VAS scores range from 0 (dead) to 100 (full health). If EQ-5D results were stratified (e.g. by age, sex and disease severity), results were only reported once, using the most clinically relevant stratification: CCS angina classification; NYHA heart failure classification or demographic characteristics (% of males/females and mean age of patient cohort). Error bars in Figures 2 and 3 represent 95% confidence intervals around the mean score, which were calculated from the reported SD and sample size. There was no attempt to combine estimates from different studies in a formal meta-analysis since the main objective was to contrast studies with different features and to explain heterogeneity in the results. The degree of heterogeneity between studies was quantified using the *I*^2 ^statistic [20]. The *I*^2 ^statistic uses the sum of the squared differences of each study from the pooled estimate and the degrees of freedom of the test to provide a measure of the percent of total variation across studies due to heterogeneity between studies. A meta-analysis yielding a value of *I*^2 ^above 75% suggests a high level of heterogeneity between the studies. Psychometric properties were summarised according to the type of property assessed (validity/reliability/responsiveness), the comparison performed, and the statistical test result.

## Results

The electronic search of databases returned 147 papers of which 66 met the selection criteria. 60 publications reported an EQ-5D index score, VAS score and/or responses to the self-classification system, whilst 10 papers presented evidence of the psychometric properties of the EQ-5D (Figure 1).

Overall, there was wide variation in terms of CVD subgroups, disease stage, age distribution and other methodological aspects (Table 1 in Additional file 1). Of studies reporting mode of administration (n = 41), 42% were filled out on-site by respondents, 52% were mailed-out questionnaires, and 6% were administered via telephone interview. Overall, there was an equal mix of randomised controlled trial (RCT) and observational study designs. Prospective observational study designs were more common than retrospective (69% vs. 31%) and there was an equal mix of longitudinal and cross-sectional studies. The majority of studies (52%) reported EQ-5D index scores using the UK-based algorithm although scores based on Czech, Danish, Dutch, German, US and European preferences were also used [13-18]. However, a number of studies (33%) did not explicitly state the algorithm used to calculate the index score.

Studies of cardiovascular patients that reported psychometric properties of the EQ-5D (n = 10) explored construct validity (convergent and discriminative), typically in terms of correlations with other disease-specific HRQoL measures as well as reliability and responsiveness (Table 2 in Additional file 1). Evidence of validity and reliability were reported in studies of ischaemic heart disease (n = 3); cerebrovascular disease (n = 3); heart failure (n = 2) and peripheral vascular disease (n = 2). Convergent validity was the most common property assessed, using Spearman rank correlations to explore associations with another measure. Reliability and responsiveness were generally measured by test-retest statistics; intra-class correlation coefficients (ICC) and effect size (ES). In terms of construct validity, comparisons were made between the EQ-5D and disease specific questionnaires such as the Barthel Index (BI), Kansas City Cardiomyopathy Questionnaire (KCCQ), MacNew Heart Disease Quality of Life Questionnaire, NYHA and VascuQol as well as other generic measures such as the Health Utilities Index (HUI2; HUI3) and the RAND Short Form Health Survey (SF-36) and its derivatives (SF-6D; SF-12;).

For convergent validity, moderate to strong agreement represented as significant correlation was generally found between EQ-5D Index and VAS scores and other generic HRQoL measures both at the domain and index level [21-23]. For discriminative validity, the EQ-5D was less able to detect clinical changes than other disease specific measures such as the KCCQ or NYHA and performed better when detecting large rather than small changes in disease severity [24]. There was also evidence of strong ceiling effects (i.e. inability to discriminate between comparatively good health states) across both domain and index values [21,25]. In general, the EQ-5D Index and VAS showed good reliability and responsiveness in comparison to other generic measures such as the SF-12 but were less responsive than disease-specific measures such as the KCCQ [26,27].

A wide range of mean and median EQ-5D scores were reported (Table 3 in Additional file 1). Studies of patients with ischaemic heart diseases (ICD codes I20-I25) reported mean index scores that ranged from 0.45 (SD 0.22) to 0.88 (no SD reported). Visual analogue scale (VAS) scores ranged from a mean of 45 (SD 17) to 82 (SD 13). Studies of heart failure (I50) patients reported mean index scores ranging from 0.31 (no SD) to 0.78 (0.11) and mean VAS scores from 37 (21) to 73 (18). Studies of cerebrovascular diseases (I60-I69) reported mean index scores ranging from 0.24 (0.39) to 0.90 (0.16) and mean VAS scores from 51 (SD 20) to 89 (no SD). Studies of peripheral vascular diseases (I73) reported mean index scores ranging from 0.33 (no SD) to 0.78 (0.23) and mean VAS scores ranging from 49 (no SD) to 71 (8).

The lowest mean EQ-5D index scores were reported in female patients with intermittent claudication undergoing secondary amputation [28]; patients with a large deterioration in heart failure [24]; and post-stroke patients [29] (Table 3 in Additional file 1). The highest mean EQ-5D index scores were reported in elderly CHD patients one year after undergoing exercise training [30]; post-trans-ischaemic attack (TIA) patients at four-year follow-up [31] and patients with history of subarachnoid haemorrhage [32].

An attempt was made to stratify mean EQ-5D index or VAS scores by disease severity (for example by CCS angina grading scale or NYHA heart failure classification). Both the CCS and NYHA scales range from class I (mild symptoms) to class IV (severe symptoms) and CCS can also be graded as 0 for no symptoms (Table 4 in Additional file 1). A previously published study stratified mean EQ-5D scores across CCS grades for patients with stable angina [33]. The results showed mean EQ-5D scores decreasing as the severity of angina increased. EQ-5D index scores ranged from 0.36 (95% CI: 0.25 to 0.48) for CCS grade IV to 0.81 (95% CI: 0.77 to 0.85) for CCS grade 0. Here, there was sufficient data available to stratify mean EQ-5D index scores by NYHA class in heart failure patients and by CCS class in patients with ischaemic heart disease (IHD). EQ-5D index scores were stratified into three categories of NYHA or CCS class based on the percentage of patients in a given group in a study in class III/IV (0-33%; 34-67%; 68-100%). It was assumed here that 0-33% in class III/IV corresponds to mild HF/angina whilst 68-100% corresponds to moderate/severe HF/angina.

In almost all cases, mean EQ-5D index scores increased with an increase in the proportion of patients with mild disease (Figure 2). Mean EQ-5D index scores decreased from 0.78 (SD 0.18) for mild states to 0.51 (SD 0.21) for moderate/severe health states. In common with heart failure patients, mean EQ-5D index scores for IHD patients generally decreased with the increasing proportions of patients with moderate/severe angina (Figure 3). Here, scores decreased from a mean of 0.80 (SD 0.05) for mild angina to 0.45 (SD 0.22) for moderate/severe angina.

An initial attempt was made to summarise the burden of CVD for each disease subgroup by calculating pooled means across studies. Both fixed and random effects meta-analyses were carried out for studies that used reported EQ-5D index scores and disease severity in terms of either CCS Angina class or NYHA heart failure class at baseline. Fixed and random effects meta-analyses of heart failure patients stratified by NYHA class and IHD patients stratified by CCS angina class produced *I*^2 ^indices of between 82-96%, suggesting a high level of statistical heterogeneity between studies [34]. Such a degree of heterogeneity between studies ruled out any further estimation of pooled mean utility scores according to disease severity.

**Figure 4:**
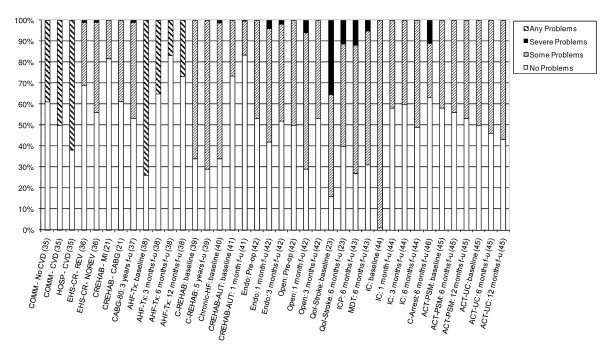
**Distribution of Scores for Mobility Dimension of EQ-5D**.

**Figure 5:**
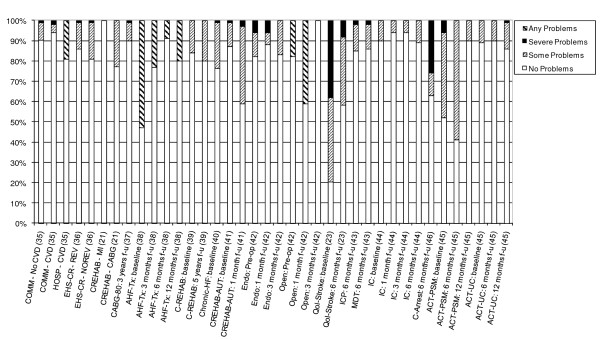
**Distribution of Scores for Self-Care Dimension of EQ-5D**.

**Figure 6:**
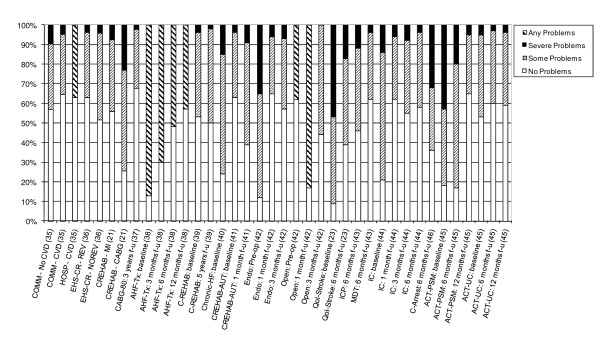
**Distribution of Scores for Usual Activities Dimension of EQ-5D**.

**Figure 7:**
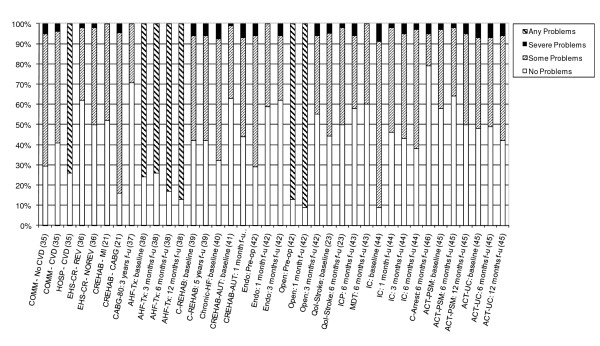
**Distribution of Scores for Pain/Discomfort Dimension of EQ-5D**.

**Figure 8:**
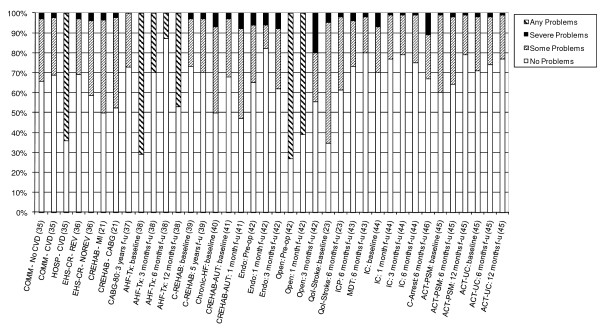
**Distribution of Scores for Anxiety/Depression Dimension of EQ-5D**.

14 studies also provided detailed information on the dimension-specific burden of cardiovascular disease, exploring the distribution of scores across the five dimensions of the EQ-5D [21,23,35-46]. In examining the dimension-specific burden of disease among cardiovascular studies, the trend in the distribution of scores was fairly similar across all five dimensions. In general, problems with usual activities tended to be most common, followed by problems with mobility and pain/discomfort (Figures 4, 5, 6, 7 and 8).

## Discussion

In recent years use of the EQ-5D to measure patient HRQoL in published studies within the cardiovascular field has increased. This largely reflects the growing requirement, over time, of clinical trials to consider cost-effectiveness alongside the clinical effectiveness of new interventions. As the "gold standard" form of economic evaluation in many health care systems, cost-utility analyses (CUA) rely on generic measures such as the EQ-5D for the calculation of QALYs. Increased use of the EQ-5D may also support the view that patient reported outcomes and quality of life are becoming more widely accepted as routine measures in clinical studies, with the EQ-5D being an internationally recognised generic measure of HRQoL. This summary of EQ-5D index and VAS scores in the cardiovascular field complements other published reports describing the use of the EQ-5D in the cancer and asthma/COPD literature and of utility scores associated with various conditions [47-50].

The review found that the majority of studies that included the EQ-5D were within IHD (I20 - I25) and cerebrovascular disease (I60 - I69), subgroups, reflecting the relative prevalence of these diseases worldwide. Stratification by disease severity (measured by CCS angina or NYHA heart failure scales) was possible for IHD patients and heart failure patients and illustrated a positive relationship with the EQ-5D when moving from severe to mild disease severity (Figures 2 and 3). However, calculation of pooled means across studies using meta-analytic techniques was not appropriate, given the high level of heterogeneity in terms of study design and patient characteristics. In general, evaluations of the validity and reliability of the EQ-5D suggested fairly strong convergent validity when assessed by correlations with other HRQoL measures and good discriminative abilities in detecting patients whose health status changed by a given clinical magnitude. However, there was evidence of strong ceiling effects across each domain for the index values. In terms of the dimension-specific burden of cardiovascular disease, problems with pain or discomfort were the most common, followed by problems with usual activities and mobility.

There was much heterogeneity in the scores observed across the studies, which was not necessarily entirely explained by the range of cardiovascular subgroups. The diverse range of index and VAS scores was also related to stage of illness or treatment (for example baseline versus post-treatment measurements) as well as non-disease-related factors such as other co-morbidities and demographic characteristics. Furthermore, no *a priori *quality criteria were imposed on studies included for review in terms of sample size or methodological quality which may explain some of the heterogeneity. On the other hand, imposing stringent inclusion criteria in terms of study methodological quality would have reduced the potential availability of studies considered for analysis. It is difficult to predict to what extent the level of heterogeneity would have been reduced if more stringent inclusion criteria had been imposed for the literature review.

Overall, this study illustrated the difficulty in attempting to adequately deal with statistical heterogeneity based on aggregated data from published studies [51]. This would suggest that individual patient-level data is required in order to estimate mean utility scores according to disease stage, at least within the cardiovascular field. Furthermore, not all studies used the same algorithm to calculate index-based scores with a third of studies also failing to report which scoring system was used. The choice of algorithm used to convert self-classification scores can affect the index-based score, as shown in a recent study which compared UK and US scoring algorithms in patients undergoing percuatenous coronary intervention (PCI) [52]. However, whilst country-specific societal preferences may reduce the scope for comparing HRQoL estimates across studies from different countries, they are more helpful to local decision making, especially when allocating resources within national health care programmes.

## Conclusion

HRQoL measures such as the EQ-5D can be useful tools to clinicians in terms of evaluating the impact of cardiovascular disease on patients and can help to inform decision making and resource allocation. The use of the EQ-5D in CVD studies has increased in recent years and published studies provide evidence of its validity and reliability. The variation in EQ-5D index and VAS scores reported here largely reflect systematic differences in terms of disease stage, treatment and patient characteristics. In the future, as more studies of CVD present EQ-5D scores according to disease severity, it may be possible to calculate pooled mean estimates that can be useful in modelling of CVD-related health outcomes in economic evaluations.

## Abbreviations used in Tables/Figures

AAA: Abdominal aortic aneurysm; ACS: Acute coronary syndromes; ACT: Anticoagulation therapy; AF: Atrial fibrillation; AH-Drug: Anti-hypertensive drug therapy; AHF: Advanced heart failure; AMI: Acute myocardial infarction; Amp.: Amputation; Angio: Coronary Angiography; ASA: American Society of Anaesthesiologists; ASCOT-AHD: Anglo-Scandinavian cardiac outcomes trial - anti-hypertensive drug treatment; Asym./Sym.: Asymptomatic/Symptomatic; AUT: Austria; AVR: Aortic valve replacement; BI: Barthel Index; BOTH-CABG/PCI/MM: Patients who are suitable for both CABG and PCI and receive CABG/PCI/MM; CABG: Coronary artery bypass graft; CABG-80: Coronary artery bypass surgery in octogenarians; CABG-CABG/PCI/MM: Patients who are suitable for CABG and receive CABG/PCI/MM; CABG-CPB: CABG using heart lung machine; CAD: Coronary artery disease; C-Arrest: Cardiac arrest; CCR: Comprehensive cardiac rehabilitation; CCS: Canadian Cardiovascular Society; CCU: Coronary care unit; CES-D: Centre for Epidemiological Studies - Depression Scale; CHD: Coronary heart disease; CHD-PHARM/Control: Community pharmacy-led medicines management programme/control treatment for patients with CHD; CML: Case method learning supported lipid-lowering strategy; COMM - CVD/NOCVD: Community dwelling-based elderly patients with/without CVD; CR: Cardiac resynchronisation; CR-Home/Hosp: Home/Hospital-based cardiac rehabilitation; C-REHAB: Cardiac rehabilitation; CS: Conservative strategy; CVA: Cerebrovascular Accident; CVD: Cardiovascular disease; Duplex US: Duplex ultrasonography; Echo: Echocardiography; EHS-CR: Euro Heart Survey on coronary revascularisation; Endo: Endovascular AAA surgery; ES: Effect size; Exercise-Qol: Long-term effects of exercise training on quality of life; F-u: Follow-up; GRS: Guyatt's responsiveness statistic; HeartMed: Lifestyle advice intervention by community pharmacists for heart failure patients; HF: Heart failure; HOSP - CVD: Hospital-based elderly patients with CVD; HRQoL: Health-related quality of life; HUI2/3: Health Utilities Index mark 2/3; IC: Intermittent claudication; ICC: Intra-class correlation; ICD: Implantable cardioverter defibrillator; ICP: Integrated care pathway; IHD: Ischaemic heart disease; IQR: Inter-quartile range; IS: Interventional strategy; IV: Intravenous; KCCQ: Kansas City Cardiomyopathy Questionnaire; LV: Left ventricular; MacNew: MacNew Heart Disease Quality of Life Questionnaire; MCS: Mental component summary; MDT: Multi-disciplinary team; MEDMAN: Community pharmacy-led medicines management services; MEPS: Medical expenditure panel survey; MI: Myocardial infarction; MI - Self-help: Home-based self-help rehabilitation package for MI patients; MIDCAB: Minimally invasive direct CABG; MM: Medical management; MR Angio: Magnetic resonance angiography; MRI: Magnetic resonance imaging; MT: Medical therapy; MVPS: Mitral valve prolapse syndrome; NYHA: New York Heart Association; OP-CABG: Off-pump CABG; Open: Open AAA surgery; PAOD: Peripheral arterial occlusive disease; PCI: Percutaneous coronary intervention; PCI-BMS: PCI with bare-metal stents; PCI-CABG/MM/PCI: Patients who are suitable for PCI and receive CABG/PCI/MM; PCI-DES: PCI with drug-eluting stents; PCS: Physical component summary; PER: Peripheral endovascular revascularisation; Pre-op: Pre-operation; Proxy: HRQol questionnaire completed by spouse/family member; PSM: Patient self-management; P-PTCA: Primary PTCA; P-Stent: Primary stent placement; PTCA: Percutaneous transluminal coronary angioplasty; QLMI: Quality of Life after MI questionnaire; QoL: Quality of life; RCT: Randomised controlled trial; REV/NO REV: Eligible/Ineligible for Revascularisation; SAH: Subarachnoid haemorrhage; SCOPE-Drug/Control: Study on cognition and prognosis in the elderly - Drug/Control treatment; SD: Standard deviation; SES: Socioeconomic status; SF-36: Short-form 36-item health survey questionnaire; SF-6D: Short-form 6D; SF-12: Short-form 12-item health survey questionnaire; SPECT: Single photon emission computed tomography; SRM: Standardised response means; Stroke-4Y: Four years post-stroke; TIA: Trans-ischaemic attack; Trans.: Heart Transplantation; Tx: Treatment; UC: Usual care; VAD: Ventricular assist device; VAS: Visual analogue scale; VascuQol: Vascular Quality of Life Questionnaire; -ve/+ve: Deterioration/Improvement in condition; WHO-ICD: World Health Organisation - International Classification of Diseases; W-list: Waiting-list.

## Competing interests

The authors declare that they have no competing interests.

## Authors' contributions

MD participated in the design of the study, carried out the systematic literature review, conducted any data analysis and drafted the manuscript. KG provided support in the statistical analysis and helped to draft the manuscript. LS participated in the design of the study, provided support in the statistical analysis and helped to draft the manuscript. MB conceived of the study, participated in the design of the study and helped to draft the manuscript. All authors read and approved the final manuscript.

## Supplementary Material

Additional file 1 **Tables**. Table 1: Description of studies that have used the EQ-5D as an outcome measure in clinical and observational studies of patients with cardiovascular disease. Table 2: Summary of studies examining validity and reliability of EQ-5D in cardiovascular disease (n = 10). Table 3: Summary of EQ-5D utility scores reported in cardiovascular studies. Table 4: Canadian Cardiovascular Society (CCS) and New York Heart Association (NYHA) classification systems [53-95]. Click here for file
