# Supplementary material for: A review of health utilities using the EQ-5D in studies of cardiovascular disease
Source: Health Qual Life Outcomes. 2010 Jan 28;8:13. doi: 10.1186/1477-7525-8-13 (PMC2824714; doi:10.1186/1477-7525-8-13)
Supplement: Additional file 1 — Tables. Table 1: Description of studies that have used the EQ-5D as an outcome measure in clinical and observational studies of patients with cardiovascular disease. Table 2: Summary of studies examining validity and reliability of EQ-5D in cardiovascular disease (n = 10). Table 3: Summary of EQ-5D utility scores reported in cardiovascular studies. Table 4: Canadian Cardiovascular Society (CCS) and New York Heart Association (NYHA) classification systems [53-95]. [file 1477-7525-8-13-S1.DOC]

**Table 1: Description of studies that have used the EQ-5D as an outcome measure in clinical and observational studies of patients with cardiovascular disease**

| **CVD Subgroup (ICD code)/ Study reference** | **Study design** | **Disease/ treatment stage (Baseline)** | **Study information** | **Patient subgroup(s)** | **Male/**  **Female (%)** | **Mean Age at Baseline (SD) [range]** |
| --- | --- | --- | --- | --- | --- | --- |
| **Ischaemic Heart Diseases (I20 – I25)** | | | | | | |
| Ascione et al. (2004) (1) | RCT | CCS: 50-59% Class III/IV; 41-50% Class I/II | Randomised to off-pump CABG (OP-CABG) or CABG using a heart-lung machine (CABG-CPB) | Patients with previous MI or requirement for bypass surgery | 80/20 | CABG-CPB  61 (11)  OPCAB  63 (9) |
| Borowiak et al. (2006) (2) | Prospective cross-sectional | N/A | Evaluates HRQoL impact of chronic CVD and hospitalisation in community-dwelling elderly | Elderly with CVD; elderly without CVD; hospitalised cardiac patients | 33/67 | 74 (6) |
| Brunner-La Rocca et al. (2007) (3) | RCT | N/A | Randomised to PCI with drug-eluting stents (DES) or PCI with bare-metal stents (BMS) | All patients receiving PCI, irrespective of indication | DES 78/22  BMS 79/21 | 64 (11) |
| Denvir et al. (2006) (4) | Prospective longitudinal | CCS: 47-55% Class III/IV; 45-53% Class I/II | Explores influence of socioeconomic status (SES) on HRQoL post-PCI | Patients undergoing PCI | High SES  69/31  Low SES  63/37 | 62 (10)  59 (10) |
| Dunning et al. (2008) (5) | Prospective cross-sectional | CCS: 71% Class III/IV; 29% Class I/II | Evaluates HRQoL ten years post-CABG surgery | Patients undergoing CABG | 77/23 | 61 |
| Eefting et al. (2003) (6) | RCT | CCS: 39-53% Class III/IV; 16-27% Class I/II | Randomised to PCI with stenting or off-pump bypass surgery | Patients referred for angioplasty | Stent 70/30  Off-pump72/28 | 60 (9)  59 (10) |
| Griffin et al. (2007) (7) | Prospective longitudinal | CCS: 67-70% Class III/IV; 25-27% Class I/II | Patients received CABG; PCI or MM | Patients with chronic coronary disease | 77/23 | 59 (9) |
| Hage et al. (2003) (8) | RCT | N/A | Randomised to either three-month period of aerobic group training three times a week or control | Elderly patients admitted to CCU due to acute coronary event (MI or unstable angina) | 80/20 | 71 [65-84] |
| Kiessling et al. (2005) (9) | RCT | N/A | Randomised to case method learning (CML) – supported lipid-lowering strategy versus control | Patients requiring secondary prevention of CAD | 78/22 | 60 (8) |
| Kim et al. (2005) (10) | RCT | CCS: 36% Class III/IV | Randomised to early interventional strategy (IS) versus conservative strategy (CS) | Patients experiencing chest pain at rest with previous evidence of CAD. | IS 61/39  CS 64/36 | 63 (10)  62 (11) |
| Kruse et al. (2006) (11) | RCT | N/A | Randomised to comprehensive cardiac rehabilitation or usual care | Patients with CHD | N/A | N/A |
| Lacey et al. (2004) (12) | Retrospective longitudinal | N/A | Intervention group received self-help plus standard care whilst control group received standard care alone | Post-MI patients in deprived area of UK | 66/34 | 68 (12) |
| Legrand et al. (2004) (13) | RCT | CCS (Stable Angina): 57-60% | Randomised to undergo stent implantation or CABG | Patients with multivessel disease | Stent 77/23  CABG 76/24 | 61 (10)  61 (9) |
| Lenzen et al. (2006) (14) | Retrospective cross-sectional | N/A | Compared HRQoL over 1-year of patients who were either eligible or ineligible for revascularisation | Patients diagnosed with CAD | 76/24 | 63 (11) |
| MEDMAN study (2007) (15) | RCT | N/A | Randomised to initial consultation with community pharmacist or standard care | Patients with CHD (previous MI, angina, CABG and/or Angioplasty) | 67/33 | 69 (9) |
| Puskas et al. (2004) (16) | RCT | CCS: 12-25% Class III/IV | Randomised to off-pump (OP-CABG) versus conventional CABG revascularisation (CABG-CPB) | Patients unselected for coronary anatomy, ventricular function or co-morbidities | OP-CABG 78/22  CABG-CPB  77/23 | 62 (11)  63 (9) |
| Reeves et al. (2004) (17) | RCT | NYHA: 16-41% Class III/IV; 59-84% Class I/II | Randomised to minimally invasive direct CABG (MIDCAB) or PTCA with stenting | Patients with proximal stenosis of the left anterior descending coronary artery | MIDCAB 70/30  PTCA  86/14 | 59 (53-67)  55 (49-61)  Median (IQR) |
| Schweikert et al. (2006) (18) | Prospective cross-sectional | NYHA: 86% Class I; 9% Class II | Analysis of psychometric properties of EQ-5D questionnaire | Patients with ACS undergoing cardiac rehabilitation | 85/15 | 55 (8) |
| Sharples et al. (2007) (19) | RCT | CCS: 11-16% Class III/IV; 78-84% Class I/II | Patients randomised to one of four initial diagnostic tests: 1) Angio; 2) SPECT; 3) cardiac MRI; 4) Stress Echo | Patients with suspected or known CAD | 67-71/29-33 | 62 (10) |
| Shrive et al. (2005) (20) | Prospective longitudinal | N/A | Economic evaluation comparing sirolimus-eluting stents with conventional stents | Patients undergoing PCI | 71/29 | 62 (12) |
| Sollano et al. (1998) (21) | Retrospective cross-sectional | N/A | Patients managed with surgery (CABG) or medical management | Octogenarians with significant multi-vessel CAD | 57/43 | 82 (2) |
| Taylor et al. (2007) (22) | RCT | N/A | Patients randomised to home-based (nurse-led) or hospital-based rehabilitation for 8-10 weeks | Patients with uncomplicated AMI and without major co-morbidity | 81/19 | 62 (11) |
| Xie et al. (2008) (23) | Retrospective cross-sectional | N/A | 2000 and 2002 MEPS data used to evaluate impact of CHD on HRQoL in the US | Patients with CHD | 56/44 | N/A |
| **Heart Failure (I50)** | | | | | | |
| Almenar-Pertejo et al. (2006) (24) | Prospective longitudinal | N/A | Evaluates HRQoL over 12 months in patients with advanced HF | Patients with advanced HF on waiting list for heart transplant | 82/18 | 53 (2) |
| Austin et al. (2008) (25) | RCT | NYHA: 56% Class II; 44% Class III | Patients randomised to cardiac rehabilitation or standard care | Patients over 60; NYHA II or III; and left ventricular systolic dysfunction | 67/33 | 72 |
| Calvert et al. (2005) (26) | RCT | NYHA: 94% Class III; 6% Class IV | HRQoL of patients with chronic HF at baseline phase | Patients with heart failure due to LV dysfunction | 74/26 | 65 (10) |
| Cleland et al. (2005) (27) | RCT | NYHA: 7% Class IV | Randomised to receive medical therapy (MT) alone or with cardiac re-synchronisation (CR) | Patients with NYHA class III or IV heart failure due to LV dysfunction and cardiac dyssynchrony | MT 73/27  CR 74/26 | 66 (59-72)  67 (60-73)  Median (IQR) |
| Hofer et al. (2006) (28) | Prospective Longitudinal | NYHA: 96% Class I/II; 4% Class III/IV | Evaluated HRQoL over 1 month in patients with heart disease referred to cardiac rehab centres | Post-MI patients with or without revascularisation | 72/28 | 61 (13) |
| Holland et al. (2007) (29) | RCT | NYHA: 33% Class I/II; 67% Class III/IV | Randomised to receive symptom self-management and lifestyle advice by community pharmacists or usual care | Patients diagnosed with HF after emergency admission | INT 64/36  UC 63/37 | 78 (9)  76 (10) |
| Sharples et al. (2006) (30) | Prospective longitudinal | NYHA: 100% Class III/IV | Patients either implanted with ventricular assist device or on IV inotropic support prior to heart transplantation | Patients with end-stage heart failure who would not survive to transplant without mechanical circulatory support | 84/16 | 42 (13)  [16-63] |
| Spertus et al. (2005) (31) | Prospective longitudinal | NYHA: 41% Class II; 44% Class III | Monitoring study of HF outpatients across 14 centres | Outpatients diagnosed with HF | 75/25 | 61 (13) |
| **Aortic Aneurysm (I71)** | | | | | | |
| Lottman et al. (2004) (32) | RCT | ASA: 60-79% Class II; 21-40% Class III | Evaluation of HRQoL following endovascular or open AAA surgery | Patients with AAAs requiring elective treatment | Endo 95/5  Open 84/16 | 68 [52-82]  68 [52-81] |
| Spencer et al. (2004) (33) | Prospective cross-sectional | N/A | Evaluation of HRQoL 12 months post-screening for AAA | Men with suspected AAA | 100/0 | 73 (5) |
| **Atrial Fibrillation (I48) & other cardiac arrhythmias (I49)** | | | | | | |
| Buxton et al. (2006) (34) | Prospective longitudinal | NYHA: 26% Class III/IV | Implantable cardioverter defibrillator (ICD) therapy in different patient groups | Patients with atrial fibrillation (AF) | 81/19 | 60 (14) |
| Groeneveld et al. (2007) (35) | Prospective cross-sectional | N/A | ICD therapy among primary and secondary prevention patients | Patients who had previously received ICD treatment for AF | 73/27 | 60 (15) |
| van Eck et al. (2008) (36) | Prospective longitudinal | N/A | Assessment of HRQoL at 1 year in patients implanted with pacemaker | Patients with indication for pacemaker implantation | 58/42 | 73 (11) |
| **Cerebrovascular diseases (I60 – I69)** | | | | | | |
| Haacke et al. (2006) (37) | Retrospective cross-sectional | N/A | Evaluation of long-term HRQoL (4 years post-stroke) | Patients admitted to hospital following stroke | 54/46 | 75 |
| Park et al. (2005) (38) | RCT | Barthel Index: 11 | Randomised to receive 12 sessions of either real or sham acupuncture during 2 weeks | Patients with recent episode of stroke (<4weeks) | 52/48 | 75 (10) |
| Pickard et al. (2004) & Pickard et al. (2005) (39, 40) | Prospective longitudinal | BI: 53 | Evaluation of HRQoL over 6 months in post-stroke patients | Patients hospitalised after ischaemic stroke | 53/47 | 68 (15) |
| Ryan et al. (2006) (41) | RCT | BI: 16 (12-18)  Median (IQR) | Randomised to intensive versus non-intensive home-based rehabilitation provision | Elderly patients recently discharged from hospital after suffering stroke/hip fracture | 39/61 | 77 (6) |
| Sulch et al. (2002) (42) | RCT | BI: 5  Median | Randomised to receive integrated care pathway (ICP) or conventional multidisciplinary team (MDT) care | Stroke patients | ICP 46/54  MDT 56/44 | ICP 75 (11)  MDT 74 (10) |
| van der Schaaf (2006) (43) | Retrospective cross-sectional | N/A | Compared HRQoL in patients with newly detected aneurysm that was left untreated (cases) and patients with negative screening (controls) | Patients with previous subarachnoid haemorrhage | Cases 29/71  Controls 38/62 | Cases 55 [31-70]  Controls 56 [29-67] |
| Xie et al. (2006) (44) | Retrospective cross-sectional | N/A | 2000 and 2002 MEPS data used to evaluate impact of stroke on HRQoL in the US | Stroke patients | 44/56 | N/A |
| **Hypertensive diseases (I10 – I15)** | | | | | | |
| Degl’Innocenti et al. (2004) (45) | RCT | N/A | Randomised to receive candesartan (anti-hypertensive) or control | Elderly patients with hypertension | 64/36 | 76 |
| Krijnen et al. (2005) (46) | RCT | N/A | Randomised to balloon angioplasty or anti-hypertensive drug therapy | Patients with hypertension and renal artery stenosis | Angio 66/34  AH-Drug 56/34 | 59 (10)  61 (10) |
| Lindgren et al. (2007) (47) | RCT | N/A | Randomised to lipid-lowering treatment or placebo | Patients with mild to moderate hypertension | Lipid 72/28  Placebo 72/28 | 69  65 |
| **Peripheral vascular diseases (I73)** | | | | | | |
| Cook & Galland (1997) (48) | Prospective longitudinal | N/A | HRQoL assessed one year post PTCA | Patients with intermittent claudication | 50/50 | 65 |
| De Vries et al. (2006) (49) | RCT | RC: 89% 1-4; 11% 5-7 | Randomised to duplex ultrasonography or MR angiography | Patients diagnosed with PAD | Duplex 70/30  MR 66/34 | 65 (11)  65 (11) |
| Safley et al. (2007) (50) | Prospective longitudinal | N/A | HRQoL assessment of patients undergoing peripheral endovascular revascularisation | Patients diagnosed with PAD | 62/38 | 68 (11) |
| Slovacek et al. (2007) (51) | Prospective longitudinal | N/A | Patients treated with endovascular intervention using percutaneous transluminal balloon angioplasty | Patients with peripheral arterial occlusive disease (PAOD) | 67/33 | 63 [49-79] |
| Tangelder et al. (1999) (28) | RCT | N/A | Randomised to oral anticoagulants or aspirin after infrainguinal bypass grafting surgery | Patients with intermittent claudication or treated for critical ischaemia | 64/36 | 70 |
| **Other/Unclassifiable** | | | | | | |
| Bosch et al. (1999) & Bosch & Hunink (2000) (52, 53)  **(Arterial embolism & Thrombosis I74)** | RCT | N/A | Randomised to primary stent placement versus primary angioplasty followed by selective stent placement | Patients with iliac artery occlusive disease | Stent 71/29  PTCA 73/27 | 60 (10)  59 (11) |
| Burstrom et al. (2001) (54)  **(I10-I15 & I20-I25)** | Retrospective cross-sectional | N/A | Cross-sectional public health survey of HRQoL among representative sample of Swedish population | Respondents with self-reported angina or hypertension | 46/54 | 49 |
| Granja et al. (2002) (55)  **(Cardiac Arrest I46)** | Prospective cross-sectional | N/A | Evaluates HRQoL of cardiac arrest survivors 6-months post-discharge | Cardiac arrest survivors | 63/37 | 47 (22-79)  Median (IQR) |
| Hung et al. (2007) (56)  **(Mitral valve disorders I34)** | RCT | N/A | HRQoL impact of programmed exercise programme versus control in female patients | Female patients with history of mitral valve prolapse syndrome (MVPS) | 0/100 | Exercise 40  Control 34 |
| Jowett et al. (2006) (57) | RCT | N/A | Patient self-management (PSM) versus usual clinic-based care (UC) of anticoagulation therapy | Patients with long-term indication for oral anticoagulation | 65/35 | PSM 64  UC 66 |
| Maliwa et al. (2003) (58)  **(Non-rheumatic aortic valve disorders I35)** | Retrospective cross-sectional | NYHA: 2.09 (Mean Class) | Evaluation of HRQoL of long-term AVR survivors (mean 30 years) | Long-term mechanical AVR survivors | 81/19 | 41 (12) |
| Spiraki et al. (2008) (59)  **(I20-I25 & I50)** | Prospective longitudinal | N/A | Patients admitted to single hospital with CHD or heart failure (HF) | Patients with CHD or HF | CHD 60/40  HF 57/43 | N/A |

**Table 2: Summary of studies examining validity and reliability of EQ-5D in cardiovascular disease (n=10)**

| **Author, Year** | **Cardiovascular subgroup** | **Validity** | **Reliability/Responsiveness** |
| --- | --- | --- | --- |
| **Schweikert et al. (2006) (18)** | Ischaemic Heart Diseases | Convergent: Spearman rank correlations between EQ-5D and SF-36 subscales and disease-specific MacNew questionnaire. Strong correlations ranging from 0.57-0.74 between EQ-5D Index and SF-36 subscales and 0.69-0.78 between Index and MacNew subscales (p<0.01). Moderate to strong correlations between VAS and SF-36 subscales (0.21-0.69) and MacNew subscales (0.52-0.72) (p<0.01).  Discriminative: Substantial ceiling effects for EQ-5D Index. | Reliability: Test-retest statistics used to measure rate of agreement of responses between admission and discharge (period 1) and discharge and 3-months (period 2). Reliability was generally good: mean rate of agreement was 82% in period 1 and 78% in period 2. Intraclass correlation coefficient (ICC): EQ-5D VAS was 0.82 period 1 and 0.80 period 2; EQ-5D Index was 0.91 in period 1 and 0.54 in period 2.  Responsiveness: Effect size (ES) calculated for patients with improved health states. ES in both periods higher for VAS: ES for VAS was 0.82 period 1; 0.4 period 2 and for EQ-5D Index was 0.74 period 1; 0.09 period 2. |
| **van Stel & Buskens (2006) (60)** | Ischaemic Heart Diseases | Convergent: Spearman rank correlations between utility scores and between domains of both EQ-5D and SF-6D. Correlation between the utility domains was rather diffuse with no strong correlations (>0.5) and only a few moderate correlations.  Discriminative: Ceiling effect in EQ-5D domains and index much larger than in SF-6D. | Reliability: Poor agreement between EQ-5D and SF-6D with ICC of 0.45. |
| **Nowels et al. (2005) (61)** | Ischaemic Heart Diseases | Convergent: Spearman rank correlations with SF-36 and QLMI. Strong correlations were shown between similar EQ-5D and SF-36 domains for pain (0.68); mobility (0.63) and anxiety/ depression (0.75). There was strong correlation (0.76) between the EQ-5D index and general health composite score for SF-36. Moderate to strong correlations detected between EQ-5D and QLMI domain scores: ranging between 0.34-0.56 and 0.57 for overall health.  Discriminative: compared differences in EQ-5D  Index between two groups based on their CCS category using Mann-Whitney rank-sum testing. The EQ-5D index showed excellent discrimination between patients with CCS scores from I-IV (*p <* 0.001). |  |
| **Pickard et al. (2005) (40)** | Cerebrovascular Diseases | Convergent: Spearman rank correlations between EQ-5D (Index and VAS) and HUI2: HUI3; SF-6D; BI; Modified Rankin Scale (MRS); and CES-D. Moderate to strong correlations detected between index and HUI2: HUI3; SF-6D; BI (0.45 to 0.59) and MRS and CESD (-0.30 to -0.36). Weaker correlations between VAS and HUI2: HUI3; SF-6D; BI (0.22 to 0.50) and MRS and CESD (-0.18 to -0.44). | Responsiveness: Measured by paired *t*-tests; standardised response mean (SRM); and ES. EQ-5D Index showed high responsiveness in overall sample (ES and SRM >0.80; *t* = 8.36); VAS was less responsive (ES and SRM <0.50; *t* = 3.89). |
| **Dorman et al. (1998) (62)** | Cerebrovascular Diseases |  | Reliability: Examined test-retest reliability by calculating agreement statistics using unweighted κ statistic for categorical domains and ICC for Index and VAS. Reproducibility ranged from moderate to good at domain level (κ = 0.63-0.80). Index and VAS both had excellent reproducibility (ICC = 0.86). |
| **van Exel et al. (2004) (63)** | Cerebrovascular Diseases | Convergent: Relationship between EQ-5D index and BI assessed using linear regression. Results showed that low BI scores were associated with very low EQ-5D scores (BI of 0 corresponds with EQ-5D Index of -0.25) - demonstrated the sensitivity of the EQ-5D for changes in health status. |  |
| **Eurich et al. (2006) (64)** | Heart Failure |  | Responsiveness: Guyatt’s responsiveness statistic; ES; and SRM calculated to compare responsiveness of EQ-5D with KCCQ and SF-12 between patients who changed and remained stable from baseline to 6-weeks. Overall, EQ-5D Index and VAS were less responsive than the disease-specific questionnaire (KCCQ) but showed similar responsiveness to the SF-12 PCS and MCS. |
| **Spertus et al. (2005) (31)** | Heart Failure | Discriminative: Compared responsiveness of EQ-5D with KCCQ; SF-12; and NYHA at individual patient level using *c* statistics (representing % of the time that the measure correctly identified patients with clinical change for all possible pairs of patients, one experiencing clinical change and one not. *c =* 0.5 indicates no discriminative ability and 1 indicates perfect discrimination). The EQ-5D Index and VAS *c* statistics ranged from 0.56 and 0.58 (for small clinical improvements) to 0.69 and 0.76 (for moderate to large clinical deterioration), showing less discriminative abilities than the KCCQ and NYHA but similar discriminative abilities to SF-12 PCS and MCS. |  |
| **Bosch & Hunink (2000) (53)** | Arterial embolism and Thrombosis | Convergent: ICCs of changes over time were calculated between EQ-5D and HUI3 and SF-36. Overall there was poor correlation between EQ-5D and HUI3 and SF-36 measures. ICC between EQ-5D and HUI3 was 0.33 (p<0.01) and ranged from 0.22 to 0.43 between EQ-5D and SF-36 domains. |  |
| **de Vries et al. (2005) (65)** | Peripheral vascular diseases | Construct: Cross-sectional and longitudinal construct validity compared between EQ-5D and disease-specific VascuQol. Receiver operating characteristic curves and areas under the curves were calculated to evaluate ability to discriminate between severe and mild disease at baseline and between large and small changes at follow-up. EQ-5D performed equally well as VascuQol in discriminating severe from mild disease at baseline but VascuQol was better in discriminating a large versus a small change in disease severity at follow-up. |  |

**Table 3: Summary of EQ-5D utility scores reported in cardiovascular studies**

| **CVD subgroup (ICD code)/ Reference** | **n** | **Index mean (SD)** | **VAS mean (SD)** | **Patients reporting problems on each domain (%)ª** | | | | | | **Scoring algorithm** |
| --- | --- | --- | --- | --- | --- | --- | --- | --- | --- | --- |
|  |  |  |  | **Mobility** | **Self-care** | **Usual activities** | **Pain/**  **Discomfort** | **Anxiety/**  **Depression** | |  |
| **Ischaemic Heart Diseases (I20 – I25)** | | | | | | | | | | |
| **Ascione et al. (1)**  CABG-CPB: 3 years f-u  OPCAB: 3 years f-u | 151  164 | 0.82 (0.25)  0.81 (0.24) | 77 (19)  76 (16) |  |  |  |  |  | | N/R |
| **Borowiak et al. (2)**  COMM - NO CVD  COMM – CVD  HOSP - CVD | 100  100  100 |  | 57 (21)  61 (20)  45 (17) | 1 – 61  2/3 - 39  1 – 50  2/3 – 50  1 – 38  2/3 – 62 | 1 – 81  2/3 – 19  1 – 82  2/3 – 18  1 – 59  2/3 – 41 | 1 – 63  2/3 – 37  1 – 62  2/3 – 38  1 – 17  2/3 – 83 | 1 – 26  2/3 – 74  1 – 13  2/3 – 87  1 – 27  2/3 – 73 | 1 – 36  2/3 – 64  1 – 27  2/3 – 73  1 – 39  2/3 – 61 | | UK (66) |
| **Brunner-La Rocca et al. (3)**  PCI-DES: 6 months f-u  PCI-DES: 18 months f-u  PCI-BMS: 6 months f-u  PCI-BMS: 18 months f-u | 463  463  239  239 | 0.99 (0.87-1.00)  0.99 (0.83-1.00)  0.90 (0.79-1.00)  0.89 (0.79-1.00) † |  |  |  |  |  |  | | Germany  (67) |
| **Denvir et al. (4)**  High SES: baseline  High SES: 12 months f-u  Low SES: baseline  Low SES: 12 months f-u | 876  876  462  462 | 0.55 (0.31)  0.75 (0.26)  0.47 (0.35)  0.63 (0.30) | 58 (22)  72 (19)  60 (20)  64 (21) |  |  |  |  |  | | N/R |
| **Dunning et al. (5)**  CABG: 10 years f-u | 621 | 0.70 (0.31) |  |  |  |  |  |  | | UK |
| **Eefting et al. (6)**  Stent: baseline  Stent: 1 month f-u  Stent: 6 months f-u  Stent: 12 months f-u  OP-CABG: baseline  OP-CABG: 1 month f-u  OP-CABG: 6 months f-u  OP-CABG: 12 months f-u | 131  136 | 0.64  0.80  0.84  0.82  0.65  0.69  0.83  0.83 |  |  |  |  |  |  | | N/R |
| **Griffin et al. (7)**  CABG-CABG: baseline  CABG-CABG: 6 years f-u  CABG-PCI: baseline  CABG-PCI: 6 years f-u  CABG-MM: baseline  CABG-MM: 6 years f-u  BOTH-CABG: baseline  BOTH-CABG: 6 years f-u  BOTH-PCI: baseline  BOTH-PCI: 6 years f-u  BOTH-MM: baseline  BOTH-MM: 6 years f-u  PCI-CABG: baseline  PCI-CABG: 6 years f-u  PCI-PCI: baseline  PCI-PCI: 6 years f-u  PCI-MM: baseline  PCI-MM: 6 years f-u | 281  264  35  35  262  219  94  100  120  108  145  131  33  28  96  100  148  129 | 0.54 (0.23)  0.69 (0.29)  0.48 (0.22)  0.61 (0.36)  0.60 (0.22)  0.67 (0.31)  0.45 (0.22)  0.66 (0.31)  0.50 (0.22)  0.65 (0.30)  0.54 (0.24)  0.69 (0.28)  0.56 (0.24)  0.69 (0.28)  0.57 (0.23)  0.65 (0.29)  0.61 (0.21)  0.66 (0.29) |  |  |  |  |  |  | | UK |
| **Hage et al. (8)**  Exercise-Qol: baseline  Exercise-Qol: 3 months f-u  Exercise-Qol: 12 months f-u  Control-Qol: baseline  Control-Qol: 3 months f-u  Control-Qol: 12 months f-u | 44  44  44  44  44  44 | 0.80  0.85  0.88  0.83  0.84  0.84 |  |  |  |  |  |  | | UK |
| **Kiessling et al. (9)**  CML-CAD: baseline  CML-CAD: 2 years f-u | 45  45 | 0.80 (0.17)  0.80 (0.17) |  |  |  |  |  |  | | UK |
| **Kim et al. (10)**  IS: baseline  IS: 4 months f-u  IS: 12 months f-u  CS: baseline  CS: 4 months f-u  CS: 12 months f-u | 897  839  805  912  853  820 | 0.67  0.75 (0.26)  0.75 (0.27)  0.67  0.71 (0.29)  0.74 (0.29) |  |  |  |  |  |  | | UK |
| **Kruse et al. (11)**  CCR: 12 months f-u | 68 | 0.87 | 71 |  |  |  |  |  | | Denmark  (68) |
| **Lacey et al. (12)**  MI – Self-help: baseline  MI – Self-help: 3 months f-u | 61  48 | 0.54  0.69 |  |  |  |  |  |  | | N/R |
| **Legrand et al. (13)**  Stent: 1 year f-u  Stent: 3 years f-u  CABG: 1 year f-u  CABG: 3 years f-u | 600  600  605  605 | 0.86 (0.16)  0.85 (0.17)  0.85 (0.17)  0.86 (0.17) | 78 (15)  80 (15)  76 (16)  78 (16) |  |  |  |  |  | | UK |
| **Lenzen et al. (14)**  EHS-CR – REV  EHS-CR - NOREV | 3,109  504 | 0.85 (0.69-1.00)†  0.76 (0.62-1.00) | 70 (60-80)†  65 (50-80)† | 1 – 69  2 – 30  3 – 1  1 – 56  2 – 43  3 – 1 | 1 – 86  2 – 13  3 – 1  1 – 81  2 – 18  3 – 1 | 1 – 63  2 – 33  3 – 4  1 – 51  2 – 44  3 – 5 | 1 – 62  2 – 36  3 – 2  1 – 50  2 – 48  3 – 2 | 1 – 69  2 – 28  3 – 3  1 – 59  2 – 38  3 – 4 | | UK |
| **MEDMAN study (15)**  CHD-PHARM: baseline  CHD-PHARM: 12 months f-u  CHD-Control: baseline  CHD-Control: 12 months f-u | 810  712  422  373 | 0.73 (0.60-0.80)†  0.73 (0.70-0.90)  0.73 (0.60-0.90)  0.73 (0.70-0.90) |  |  |  |  |  |  | | N/R |
| **Puskas et al. (16)**  OP-CABG: baseline  OP-CABG: 1 year f-u  CABG: baseline  CABG: 1 year f-u | 97  77  98  80 | 0.73 (0.25)  0.79 (0.28)  0.79 (0.23)  0.80 (0.26) | 60 (20)  74 (22)  62 (19)  77 (20) |  |  |  |  |  | | UK |
| **Reeves et al. (17)**  PTCA: baseline  PTCA: 3 months f-u  PTCA: 6 months f-u  PTCA: 12 months f-u  MID-CABG: baseline  MID-CABG: 3 months f-u  MID-CABG: 6 months f-u  MID-CABG: 12 months f-u | 50  43  39  38  50  41  43  40 | 0.66 (0.26)  0.79 (0.24)  0.78 (0.24)  0.77 (0.25)  0.72 (0.22)  0.84 (0.14)  0.80 (0.19)  0.82 (0.22) | 57 (20)  72 (18)  74 (20)  75 (22)  64 (17)  80 (17)  80 (14)  82 (13) |  |  |  |  |  | | UK |
| **Schweikert et al. (18)**  CREHAB – MI  CREHAB - CABG | 54  44 | 0.78  0.65 | 70  57 | 1 – 81  2 – 19  3 – 0  1 – 61  2 – 39  3 – 0 | 1 – 100  2 – 0  3 – 0  1 – 77  2 – 23  3 – 0 | 1 – 56  2 – 37  3 – 7  1 – 26  2 – 51  3 – 23 | 1 – 52  2 – 48  3 – 0  1 – 16  2 – 80  3 – 4 | 1 – 50  2 – 46  3 – 4  1 – 52  2 – 46  3 – 2 | | Europe (69) |
| **Sharples et al. (2007) (19)**  Angio: baseline  Angio: 6 months f-u  Angio: 18 months f-u  SPECT: baseline  SPECT: 6 months f-u  SPECT: 18 months f-u  MRI: baseline  MRI: 6 months f-u  MRI: 18 months f-u  Echo: baseline  Echo: 6 months f-u  Echo: 18 months f-u | 222  222  222  224  224  224  226  226  226  226  226  226 | 0.76 (0.23)  0.78 (0.24)  0.78 (0.25)  0.78 (0.19)  0.81 (0.19)  0.80 (0.20)  0.75 (0.23)  0.80 (0.22)  0.77 (0.27)  0.77 (0.22)  0.81 (0.20)  0.82 (0.21) |  |  |  |  |  |  | | UK |
| **Shrive et al. (20)**  Event Free: baseline  Cardiac Event.: baseline | 7,334  7,334 | 0.77  0.85 |  |  |  |  |  |  | | N/R |
| **Sollano et al. (21)**  CABG-80: 3 years f-u | 176 | 0.83 |  | 1 - 53  2 – 46  3 – 1 | 1 – 90  2 – 9  3 – 1 | 1 – 68  2 – 30  3 – 2 | 1 – 70  2 – 30  3 – 0 | 1 – 73  2 – 27  3 – 0 | | UK |
| **Taylor et al. (22)**  CR-Home: baseline  CR-Home: 9 months f-u  CR-Hosp: baseline  CR-Hosp: 9 months f-u | 60  48  44  32 | 0.76 (0.15)  0.74 (0.21)  0.74 (0.20)  0.78 (0.23) |  |  |  |  |  |  | | UK |
| **Xie et al. (2008) (23)**  MEPS - IHD | 2,091 | 0.73 (0.46) | 63 (27) |  |  |  |  |  | | US (70) |
| **Heart Failure (I50)** | | | | | | | | | | |
| **Almenar-Pertejo et al. (24)**  AHF-Tx: baseline  AHF-Tx: 3 months f-u  AHF-Tx: 6 months f-u  AHF-Tx: 12 months f-u | 38  27  23  15 |  | 37 (21)  68 (14)  71 (13)  66 (16) | 1 – 26  2/3 - 74  1 – 65  2/3 – 35  1 – 83  2/3 – 17  1 – 73  2/3 - 27 | 1 – 47  2/3 – 53  1 – 77  2/3 – 23  1 – 91  2/3 – 9  1 – 80  2/3 - 20 | 1 – 24  2/3 – 76  1 – 26  2/3 – 74  1 – 17  2/3 – 83  1 – 13  2/3 - 87 | 1 – 13  2/3 – 87  1 – 30  2/3 – 70  1 – 48  2/3 – 52  1 – 47  2 - 53 | | 1 – 29  2 – 71  1 – 70  2/3 – 30  1 – 87  2 – 13  1 – 53  2 – 47 | N/R |
| **Austin et al. (25)**  C-REHAB: baseline  C-REHAB: 5 years f-u | 57  57 | 0.69 (0.23)  0.61 (0.32) | 64 (18)  64 (19) | 1 – 34  2 – 66  3 – 0  1 – 29  2 – 71  3 – 0 | 1 – 82  2 – 18  3 – 0  1 – 77  2 – 23  3 – 0 | 1 – 34  2 – 61  3 – 5  1 – 32  2 – 55  3 – 13 | 1 – 41  2 – 54  3 – 5  1 – 38  2 – 48  3 – 14 | | 1 – 55  2 – 43  3 – 2  1 – 64  2 – 33  3 – 3 | N/R |
| **Calvert et al. (26)**  Chronic-HF: baseline | 740 | 0.60 (0.58-0.62) |  | 1 – 34  2 – 65  3 – 1 | 1 – 76  2 – 23  3 – 1 | 1 – 24  2 – 61  3 – 15 | 1 – 32  2 – 60  3 – 8 | | 1 – 50  2 – 43  3 – 7 | UK |
| **Cleland et al. (27)**  CR: 3 months f-u  MT: 3 month f-u | 409  404 | 0.70 (0.28)  0.63 (0.29) |  |  |  |  |  | |  | N/R |
| **Hofer et al. (28)**  C-REHAB-AUT: baseline  C-REHAB-AUT: 1 month f-u | 301  301 |  | 63 (19)  73 (18) | 1 – 74  2 – 26  3 – 0  1 – 83  2 – 16  3 – 1 | 1 – 90  2 – 9  3 – 1  1 – 94  2 – 4  3 – 2 | 1 – 57  2 – 34  3 – 9  1 – 64  2 – 31  3 – 5 | 1 – 29  2 – 65  3 – 6  1 – 41  2 – 55  3 – 3 | | 1 – 66  2 – 31  3 – 3  1 – 69  2 – 29  3 – 2 | N/R |
| **Holland et al. (29)**  HeartMed: baseline  HeartMed: 3 months f-u  HeartMed: 6 months f-u  UsualCare: baseline  UsualCare: 3 months f-u  UsualCare: 6 months f-u | 147  113  108  144  100  104 | 0.58 (0.32)  0.54 (0.33)  0.58 (0.29)  0.57 (0.34)  0.51 (0.37)  0.52 (0.34) | 57 (19)  58 (21)  58 (20)  57 (20)  53 (22)  59 (20) |  |  |  |  | |  | UK |
| **Sharples et al. (2006) (30)**  VAD: 1 month f-u  VAD: 3 months f-u  VAD: 3+ months f-u  VAD-Post-Trans: 3 months f-u  VAD-Post-Trans: 3+ months f-u  Trans. w-list: 1^st^ month  Trans. w-list: 2-3 months  Trans. w-list: 3+ months  Post-Trans: 3 months f-u  Post-Trans: 3+ months f-u | 13  21  25  9  19  4  9  55  12  26 | 0.51 (0.21)  0.66 (0.14)  0.66 (0.14)  0.76 (0.13)  0.78 (0.11)  0.61 (0.07)  0.59 (0.20)  0.68 (0.16)  0.74 (0.19)  0.78 (0.18) |  |  |  |  |  | |  | UK |
| **Spertus et al. (31)**  HF: baseline  HF + large -ve: 6 weeks f-u  HF + moderate -ve: 6 weeks f-u  HF + small -ve: 6 weeks f-u  HF + large +ve: 6 weeks f-u  HF + moderate +ve: 6 weeks f-u  HF + small +ve: 6 weeks f-u  HF no change: 6 weeks f-u | 476  5  13  35  4  34  65  320 | 0.67  0.31  0.59  0.67  0.69  0.76  0.69  0.67 | 62  57  44  60  60  71  65  62 |  |  |  |  | |  | UK |
| **Aortic Aneurysm (I71)** | | | | | | | | | | |
| **Lottman et al. (32)**  Endo – Pre-op  Endo – 1 month f-u  Endo – 3 months f-u  Open – Pre-op  Open – 1 month f-u  Open – 3 months f-u | 53  52  50  18  17  17 |  | 67 (18)  68 (14)  67 (18)  61 (17)  61 (16)  61 (17) | 1 – 53  2 – 47  3 – 0  1 – 42  2 – 54  3 – 4  1 – 52  2 – 46  3 – 2  1 – 50  2 – 50  3 – 0  1- 29  2 – 65  3 – 6  1 – 53  2 – 47  3 – 0 | 1 – 83  2 – 17  3 – 0  1 – 85  2 – 13  3 – 2  1 – 86  2 – 12  3 – 2  1 – 100  2 – 0  3 – 0  1 – 82  2 – 12  3 – 6  1 – 88  2 – 6  3 – 6 | 1 – 57  2 – 36  3 – 7  1 – 46  2 – 42  3 – 12  1 – 62  2 – 34  3 – 4  1 – 44  2 – 56  3 – 0  1 – 12  2 – 53  3 – 35  1 – 65  2 – 29  3 – 6 | 1 – 62  2 – 32  3 – 6  1 – 58  2 – 36  3 – 6  1 – 60  2 – 40  3 – 0  1 – 55  2 – 39  3 – 6  1 – 29  2 – 65  3 – 6  1 – 59  2 – 41  3 – 0 | 1 – 62  2 – 30  3 – 8  1 – 73  2 – 23  3 – 4  1 – 80  2 – 18  3 – 2  1 – 50  2 – 22  3 – 18  1 – 65  2 – 29  3 – 6  1 – 82  2 – 12  3 – 6 | | Nether-lands (71) |
| **Spencer et al. (33)**  Men - AAA  Men – normal aorta  Partner – AAA  Partner – normal aorta | 120  245  94  190 | 0.83 (0.18)  0.80 (0.21)  0.81 (0.25)  0.76 (0.23) | 69 (26)  71 (25)  79 (16)  76 (17) |  |  |  |  |  | | N/R |
| **Atrial Fibrillation (I48) & other Cardiac arrhythmias (I49)** | | | | | | | | | | |
| **Buxton et al. (34)**  ICD: 1 year f-u  ICD: 2 years f-u  ICD: 3 years f-u  ICD: 4 years f-u  ICD: 5 years f-u  ICD: 6 years f-u | 42  54  43  37  19  34 | 0.78 (0.21)  0.71 (0.27)  0.78 (0.23)  0.69 (0.30)  0.74 (0.30)  0.77 (0.20) |  |  |  |  |  |  | | UK |
| **Groeneveld et al. (35)**  ICD-Primary  ICD-Secondary | 45  75 | 0.84 (0.77-1.00)†  0.84 (0.78-1.00) | 80 (61-90) †  85 (70-92) |  |  |  |  |  | | N/R |
| **van Eck et al. (36)**  Pacemaker – baseline  Pacemaker – 1 year f-u | 501  501 | 0.69  0.75 | 63  69 |  |  |  |  |  | | N/R |
| **Cerebrovascular diseases (I60 - I69)** | | | | | | | | | | |
| **Haacke et al. (37)**  Stroke-4Y: All  Stroke-4Y: MI + TIA  Stroke-4Y: TIA  Stroke-4Y: Male  Stroke-4Y: Female | 77  54  18  35  42 | 0.73 (0.32)  0.68 (0.33)  0.90 (0.16)  0.75 (0.31)  0.72 (0.32) | 56 (29)  57 (22)  78 (18)  65 (23)  58 (23) |  |  |  |  |  | | Germany |
| **Park et al. (38)**  Real-Acupuncture: 2 weeks f-u  Sham-Acupuncture: 2 weeks f-u | 56  50 | 0.64 (0.03-0.80)†  0.64 (0.09-0.71) | 60 (49-73)  50 (50-70) |  |  |  |  |  | | N/R |
| **Pickard et al. (2004) (39)**  Patient – baseline  Patient – 1 month f-u  Patient – 3 months f-u  Patient – 6 months f-u  Proxy – baseline  Proxy – 1 month f-u  Proxy – 3 months f-u  Proxy – 6 months f-u | 124  104  100  95  124  104  100  95 | 0.31 (0.38)  0.55 (0.36)  0.61 (0.30)  0.62 (0.34)  0.24 (0.39)  0.51 (0.36)  0.56 (0.30)  0.59 (0.35) | 61 (17)  64 (19)  69 (17)  70 (18)  51 (20)  64 (20)  66 (20)  69 (20) |  |  |  |  |  | | UK |
| **Pickard et al. (2005)* (40)**  Qol-Stroke: baseline  Qol-Stroke: 6 months f-u | 98  98 | 0.31 (0.38)  0.62 (0.33) | 61 (17)  70 (18) | 1 – 16  2 – 48  3 – 36  1 – 40  2 – 49  3 – 11 | 1 – 20  2 – 41  3 – 38  1 – 58  2 – 34  3 – 8 | 1 – 9  2 – 44  3 – 47  1 – 39  2 – 44  3 – 17 | 1 – 44  2 – 51  3 – 5  1 – 50  2 – 48  3 – 2 | 1 – 35  2 – 60  3 – 5  1 – 61  2 – 37  3 – 2 | | UK |
| **Ryan et al. (41)**  Non-Intensive Tx: baseline  Non-Intensive Tx: 3 months f-u  Intensive Tx: baseline  Intensive Tx: 3 months f-u | 44  32  45  35 | 0.52 (0.26-0.75)†  0.54 (0.26-0.73)  0.56 (0.28-0.72)  0.71 (0.59-0.81) | 70 (50-80)†  70 (60-81)  60 (48-66)  70 (54-80) |  |  |  |  |  | | N/R |
| **Sulch et al. (42)**  ICP – 6 months f-u  MDT – 6 months f-u | 62  59 |  |  | 1 – 27  2 – 61  3 – 12  1 – 31  2 – 64  3 – 5 | 1 – 52  2 – 42  3 – 6  1 – 41  2 – 59  3 – 0 | 1 – 18  2 – 39  3 – 43  1 – 17  2 – 63  3 – 20 | 1 – 58  2 – 39  3 – 3  1 – 64  2 – 34  3 – 2 | 1 – 60  2 – 39  3 – 1  1 – 64  2 – 34  3 – 2 | | N/R |
| **van der Schaaf et al. (43)**  SAH - Cases  SAH - Controls | 35  34 | 0.80  0.90 | 75  81 |  |  |  |  |  | | N/R |
| **Xie et al. (2006) (44)**  MEPS - Stroke | 1,040 | 0.69 (0.32) | 62 (26) |  |  |  |  |  | | US |
| **Hypertensive diseases (I10-I15)** | | | | | | | | | | |
| **Degl’Innocenti et al. (45)**  SCOPE-Drug: baseline  SCOPE-Drug: f-u  SCOPE-Control: baseline  SCOPE-Control: f-u | 1,428  1,327  1,422  1,322 |  | 74 (17)  71 (19)  74 (17)  70 (20) |  |  |  |  |  | | UK |
| **Krijnen et al. (46)**  PTCA: baseline  PTCA: 3 months f-u  PTCA: 12 months f-u  AH-Drug: baseline  AH-Drug: 3 months f-u  AH-Drug: 12 months f-u | 56  56  56  50  50  28 |  | 80 (16)  89  86  80 (19)  88  89 |  |  |  |  |  | | Nether-lands |
| **Lindgren et al. (47)**  ASCOT-AHD: baseline  ASCOT-AHD: 3 months f-u  ASCOT-AHD: 6 months f-u  ASCOT-AHD: 12 months f-u  Control | 58  57  56  57  354 | 0.82 (0.14)  0.73 (0.19)  0.77 (0.17)  0.74 (0.22)  0.81 (0.16) | 76  64  69  71  77 |  |  |  |  |  | | UK |
| **Peripheral vascular diseases (I73)** | | | | | | | | | | |
| **Cook & Galland (48)**  IC-PTCA: Pre-PTCA  IC-PTCA: 6 weeks f-u  IC-PTCA: 1 year f-u | 24 | 0.80 (0.55-0.90)†  0.89 (0.76-0.95)  0.80 (0.75-0.90) | 84 (74-84)  84 (84-92)  92 (82-92)  † |  |  |  |  |  | | N/R |
| **De Vries et al. (49)**  MR-Angio: baseline  MR-Angio: 2 weeks f-u  MR-Angio: 3 months f-u  MR-Angio: 6 months f-u  Duplex US: baseline  Duplex US: 2 weeks f-u  Duplex US: 3 months f-u  Duplex US: 6 months f-u | 174  169  162  157  171  165  158  153 | 0.52 (0.26)  0.48  0.56  0.57  0.55 (0.26)  0.54  0.58  0.58 |  |  |  |  |  |  | | UK |
| **Safley et al. (50)**  PER – baseline  PER – 1 year f-u | 250  250 | 0.71 (0.17)  0.78 (0.23) |  |  |  |  |  |  | | N/R |
| **Slovacek et al. (51)**  Pre-PTCA  Post-PTCA | 30  30 | 0.67 (0.09)  0.72 (0.08) | 63 (12)  71 (8) |  |  |  |  |  | | Czech Republic (72) |
| **Tangelder et al. (28);(73)**  Patient Graft - Male  Patient Graft - Female  Asym. Occlusion - Male  Asym. Occlusion - Female  Sym. Occlusion – Male  Sym. Occlusion - Female  Revascularisation – Male  Revascularisation - Female  Primary Amp. – Male  Primary Amp. – Female  Secondary Amp. – Male  Secondary Amp. – Female  CVA – Male  CVA – Female  MI – Male  MI - Female | 409§  32  65  194  36  38  16  25 | 0.71  0.63  0.67  0.59  0.60  0.52  0.61  0.53  0.51  0.43  0.41  0.33  0.48  0.40  0.59  0.51 | 70  65  70  65  63  58  67  61  65  60  61  55  55  49  58  52 |  |  |  |  |  | | UK |
| **Other/Unclassifiable** | | | | | | | | | | |
| **Bosch et al. (1999) (52)**  P-Stent: baseline  P-Stent: 1 month f-u  P-Stent: 3 months f-u  P-Stent: 12 months f-u  P-Stent: 24 months f-u  P-PTCA: baseline  P-PTCA: 1 month f-u  P-PTCA: 3 months f-u  P-PTCA: 12 months f-u  P-PTCA: 24 months f-u  **(I74)** | 143  143  143  143  143  136  136  136  136  136 | 0.46 (0.20-0.75)†  0.70 (0.15-1.00)  0.75 (0.15-1.00)  0.59 (0.19-1.00)  0.70 (0.09-1.00)  0.46 (0.15-0.75)  0.70 (0.20-1.00)  0.70 (0.20-1.00)  0.70 (0.15-1.00)  0.66 (0.15-1.00) |  |  |  |  |  |  | | UK |
| **Bosch & Hunink (2000)** (53)**  IC: baseline  IC: 1 month f-u  IC: 3 months f-u  IC: 12 months f-u  **(I74)** | 88  84  84  72 | 0.57 (0.25)  0.79 (0.23)  0.77 (0.26)  0.75 (0.21) |  | 1 – 1  2 – 99  3 – 0  1 – 58  2 – 42  3 – 0  1 – 60  2 – 40  3 – 0  1 – 49  2 – 51  3 – 0 | 1 – 90  2 – 10  3 – 0  1 – 94  2 – 6  3 – 0  1 – 94  2 – 6  3 – 0  1 – 89  2 – 11  3 – 0 | 1 – 21  2 – 65  3 – 14  1 – 62  2 – 32  3 – 0  1 – 55  2 – 37  3 – 8  1 – 58  2 – 38  3 – 4 | 1 – 9  2 – 82  3 – 9  1 – 46  2 – 52  3 – 2  1 – 43  2 – 52  3 – 5  1 – 38  2 – 59  3 – 3 | 1 – 70  2 – 23  3 – 7  1 – 77  2 – 22  3 – 1  1 – 79  2 – 20  3 – 1  1 – 75  2 – 24  3 – 1 | | UK |
| **Burstrom et al. (54)**  Hypertension  Angina  **(I10-I15 & I20-I25)** | 331  191 | 0.78 (0.18)  0.70 (0.28) | 75 (18)  69 (28) |  |  |  |  |  | | UK |
| **Granja et al. (55)**  C-Arrest: 6 months f-u  **(I46)** | 19 | 0.85 (0.28-1.00)† | 80 (30-100)† | 1 – 63  2 – 26  3 – 1 | 1 – 63  2 – 11  3 – 26 | 1 – 36  2 – 32  3 – 32 | 1 – 79  2 – 16  3 – 5 | 1 – 67  2 – 22  3 – 11 | | UK |
| **Hung et al. (56)**  Exercise – baseline  Exercise – 12 weeks f-u  Control – baseline  Control – 12 weeks f-u  **(I34)** | 18  18  21  21 |  | 69 (19)  82 (12)  71 (11)  74 (9) |  |  |  |  |  | | UK |
| **Jowett et al. (57)**  ACT - PSM: baseline  ACT - PSM: 6 months  ACT - PSM: 12 months  ACT - UC: baseline  ACT - UC: 6 months  ACT - UC: 12 months | 204  200  198  165  162  162 |  |  | 1 – 58  2 – 42  3 – 0  1 – 56  2 – 44  3 – 0  1 – 53  2 – 47  3 – 0  1 – 50  2 – 50  3 – 0  1 – 46  2 – 54  3 – 0  1 – 43  2 – 57  3 – 0 | 1 – 90  2 – 10  3 – 0  1 – 89  2 – 11  3 – 0  1 – 90  2 – 10  3 – 0  1 – 86  2 – 13  3 – 1  1 – 84  2 – 16  3 – 0  1 – 80  2 – 20  3 – 0 | 1 – 65  2 – 30  3 – 5  1 – 53  2 – 42  3 – 5  1 – 60  2 – 37  3 – 3  1 – 59  2 – 37  3 – 4  1 – 53  2 – 43  3 – 4  1 – 50  2 – 48  3 – 2 | 1 – 50  2 – 45  3 – 5  1 – 48  2 – 45  3 – 7  1 – 49  2 – 44  3 – 7  1 – 42  2 – 52  3 – 6  1 – 42  2 – 52  3 – 6  1 – 42  2 – 52  3 – 6 | 1 – 79  2 – 20  3 – 1  1 – 71  2 – 27  3 – 2  1 – 74  2 – 24  3 – 2  1 – 77  2 – 22  3 – 1  1 – 73  2 – 24  3 – 3  1 – 70  2 – 27  3 – 3 | | UK |
| **Maliwa et al. (58)**  AVR: 30 years f-u  **(I35)** | 91 | 0.73 (0.24) | 68 (20) |  |  |  |  |  | | N/R |
| **Spiraki et al. (59)**  CHD: admission  CHD: discharge  CHD: 1 month f-u  HF: admission  HF: discharge  HF: 1 month f-u  **(I20-I25 & I50)** | 104  98  94  49  47  47 | 0.62  0.71  0.72  0.54  0.62  0.67 | 64  66  67  57  59  60 |  |  |  |  |  | | UK |

ª Data reported according to level of problems on each domain: 1 = no problems; 2 = some problems; 3 = extreme problems; † Median values (IQR) reported; *Based on same dataset as Pickard et al.(2004); **Based on same patient dataset as Bosch et al. (1999); *** Cross-sectional survey including patients with hypertension and angina pectoris; § total number of male and female patients combined

**Table 4. Canadian Cardiovascular Society (CCS) and New York Heart Association (NYHA) classification systems**

| Classification level | CCS | NYHA |
| --- | --- | --- |
| 0 | No symptoms | --- |
| I | Angina only with strenuous exertion or prolonged activity | No symptoms, no limitation or shortness of breath when walking or climbing stairs |
| II | Angina only during vigorous activity or under stressful circumstances, slight limitation to activities | Mild angina or shortness of breath during ordinary activity |
| III | Angina on walking 1 or 2 blocks on the level or climbing one flight of stairs , moderate limitation to activities | Marked limitation, angina when walking short distances, comfortable only at rest |
| IV | Any physical activity causes angina, may be angina at rest, severe limitation of activities | Severe limitation, angina at rest, patients often bedbound |
